# Intrinsic Immunomodulatory Effects of Low-Digestible Carbohydrates Selectively Extend Their Anti-Inflammatory Prebiotic Potentials

**DOI:** 10.1155/2015/162398

**Published:** 2015-04-21

**Authors:** Jérôme Breton, Coline Plé, Laetitia Guerin-Deremaux, Bruno Pot, Catherine Lefranc-Millot, Daniel Wils, Benoit Foligné

**Affiliations:** ^1^Bactéries Lactiques & Immunité des Muqueuses, Centre d'Infection et d'Immunité de Lille, Institut Pasteur de Lille, U1019, UMR8204, Université Lille Nord de France, 1 rue du Pr Calmette, BP 245, 59019 Lille Cedex, France; ^2^Roquette Nutritional Sciences, Roquette, 62080 Lestrem, France

## Abstract

The beneficial effects of carbohydrate-derived fibers are mainly attributed to modulation of the microbiota, increased colonic fermentation, and the production of short-chain fatty acids. We studied the direct immune responses to alimentary fibers in *in vitro* and *in vivo* models. Firstly, we evaluated the immunomodulation induced by nine different types of low-digestible fibers on human peripheral blood mononuclear cells. None of the fibers tested induced cytokine production in baseline conditions. However, only one from all fibers almost completely inhibited the production of anti- and proinflammatory cytokines induced by bacteria. Secondly, the impact of short- (five days) and long-term (three weeks) oral treatments with selected fibers was assessed in the trinitrobenzene-sulfonic acid colitis model in mice. The immunosuppressive fiber significantly reduced levels of inflammatory markers over both treatment periods, whereas a nonimmunomodulatory fiber had no effect. The two fibers did not differ in terms of the observed fermentation products and colonic microbiota after three weeks of treatment, suggesting that the anti-inflammatory action was not related to prebiotic properties. Hence, we observed a direct effect of a specific fiber on the murine immune system. This intrinsic, fiber-dependent immunomodulatory potential may extend prebiotic-mediated protection in inflammatory bowel disease.

## 1. Introduction

Inflammatory bowel diseases (IBDs) are chronic inflammatory disorders of the gastrointestinal tract that occur in genetically predisposed individuals (usually following an environmental trigger). The two major IBDs in humans are ulcerative colitis and Crohn's disease [1, 2]. Although the precise etiology of IBD remains unclear, several contributing factors have been identified. Of these, the composition of the gut microbiota has attracted much attention in the last few years [3]. Indeed, dysbiosis of the normal gut microbiota (leading to a breakdown in normal host-microbe interactions) is likely to trigger the development of IBD. One can therefore legitimately assume that modulation of the gut microbiota can have prophylactic benefits and may even be a treatment option. In humans, the microbiota is composed of four major phyla: the Firmicutes, Bacteroidetes, Actinobacteria, and Proteobacteria. In IBD, it has been reported that Bacteroidetes are less abundant and Firmicutes are less diverse [4, 5].

By modifying the diet (e.g., by adding nondigestible fibers), it is possible to modulate the gut's microbiota and the ecological environment [6]. Prebiotics are defined as “nondigestible food ingredients that beneficially affect the host by selectively stimulating the growth and/or activity of one or a limited number of bacterial species already resident in the colon and, thus, improve host health” [7, 8]. These ingredients are mainly fermented by the microbiota in the colon, where they stimulate the growth of beneficial bacteria (such as bifidobacteria and lactobacilli) and inhibit the growth of pathogenic bacteria (such as* Clostridium* species) [9]. This fermentation also leads to the production of short-chain fatty acids (SCFAs), which lowers the caecal pH and thus prevents overgrowth by pH-sensitive pathogenic bacteria (such as* E. coli* and* Salmonella *spp.) [10].

Production of the three main luminal SCFAs (acetic, butyric, and propionic acids) may have a direct impact on the immune system. Short-chain fatty acids are used directly by colonocytes as energy sources and also have well-documented anti-inflammatory properties* in vitro* and* in vivo* [11]. Although the mechanism of action is only partially understood, it has been shown that butyrate can inhibit inflammatory responses via inhibition of NF*κ*B in both peripheral blood mononuclear cells (PBMCs) and a 2,4,6-trinitrobenzene-sulfonic acid (TNBS) colitis model [12]. Acetate and propionate are less well studied but also have anti-inflammatory effects; propionate is more effective than acetate [13]. Short-chain fatty acids are known to bind to specific receptors, such as G-protein coupled receptors 41 and 43 (GPR41 and GPR43, also known as free fatty acid receptors 2 and 3 (FFAR-2 and FFAR-3), resp.). Short-chain fatty acids activate GPR41 and GPR43 on intestinal epithelial cells, leading to the rapid production of chemokines and cytokines [14]. It has been shown that GPR43 has a critical role in the recruitment of polymorphonuclear leukocytes during intestinal inflammation [15, 16]. In mice, SCFAs can regulate the size and function of the colonic regulatory T-cell pool and protect against colitis in a GPR43-dependent manner [17].

Few studies have focused on the direct effects of dietary fibers on the host (rather than indirect, postfermentation effects on promoting viability and functionality of probiotics or other colonic organisms) [18, 19]. For example, a decrease in adherence of probiotic strains to abiotic surfaces, mucus substrates, and epithelial cell lines was observed following the direct addition of benchmarked prebiotics [20]. It was recently reported that prebiotic oligosaccharides may have a direct anti-inflammatory effect. In fact, a specific carbohydrate induces peroxisome proliferator-activated receptor gamma (PPAR*γ*) and peptidoglycan recognition protein 3 (PGlyRP3), thereby inhibiting the production of inflammatory cytokines by intestinal epithelial cells [21]. Prebiotics were shown to directly activate toll-like receptor 4 (TLR4) on intestinal epithelial cells and thus induce proinflammatory responses [22]. Nevertheless, few researchers have investigated the direct effect of various types of fibers. The objective of the present study was thus to evaluate the direct, intrinsic effects of different types of fibers on the immune system by using both* in vitro* and* in vivo* models. We hypothesized that a better understanding of the mechanisms of action of low-digestible polysaccharides might prompt the development of treatments for IBD and irritable bowel syndrome that combine both prebiotic-mediated and direct effects. We differentiated between indirect and direct effects by implementing long-term and short-term treatments in a murine model of experimental colitis.

## 2. Materials and Methods

### 2.1. Prebiotic Substances Tested

Nine different types of soluble dietary fibers (referred to here as Fibers 1 to 9) were tested for their immunomodulatory effects. Fibers 1, 2, 3, 4, 5, 7, and 8 were glucose-based and Fibers 6 and 9 were fructose-based. Fibers 1 and 2 (provided by Roquette (Lestrem, France)) were manufactured by a process commonly used in the starch industry. Fibers 3 to 9 were commercially available products with construction-based polysaccharides and oligosaccharides. Glucose (Sigma, Saint-Louis, MO, USA) was used as a control.

### 2.2. Isolation and Stimulation of PBMCs

Peripheral blood mononuclear cells were isolated from the peripheral blood of healthy donors, as previously described [23]. Briefly, after Ficoll gradient centrifugation (Pharmacia, Uppsala, Sweden), mononuclear cells were collected, washed, and adjusted to 1 × 10^6^ cells/mL in RPMI complete medium (consisting of RPMI 1640 medium (Live Technologies, Paisley, Scotland) supplemented with gentamicin (150 g/mL), L-glutamine (2 mmol/L), and 10% fetal calf serum (all from Gibco-BRL, NY, USA)). The PBMCs were seeded in 24-well tissue culture plates (Corning, NY, USA). Fibers were dissolved in RPMI complete medium, filtered at 0.22 *μ*m (Millipore, Molsheim, France), and then incubated with PBMCs at a final concentration of 5 g/L.

For PBMC stimulation,* Lactococcus lactis* MG1363 and* Bifidobacterium longum* BB3001 strains were incubated alone or with fibers. The MG1363 strain was grown at 30°C in M17 broth supplemented with 0.5% glucose, and the BB3001 strain was grown at 37°C in MRS broth (Difco, Pont-de-Claix, France). Bacterial cells were grown until the stationary phase, washed, and adjusted to McFarland 3 (using a portable photometer (Densimat bioMérieux, Marcy l'étoile, France)) in PBS containing 20% glycerol and stored at −80°C until required for later assays [24]. After 24 h of stimulation at 37°C in an atmosphere of air with 5% CO_2_, culture supernatants were collected, clarified by centrifugation, and stored at −20°C until cytokine analysis. Cytokine levels were measured in an enzyme-linked immunosorbent assay (ELISA) using BD Pharmingen antibody pairs (BD Biosciences, San Jose, CA, USA) for interleukin- (IL-) 10, IL-12p70, and interferon *γ* (IFN*γ*), and from R&D Systems (Minneapolis, MN, USA) for human tumor necrosis factor alpha (TNF-*α*) and IL-6, according to the manufacturer's recommendations.

### 2.3. Animal Care and Ethical Aspects

Female BALB/c mice (6 weeks old on arrival) were obtained from Charles River Laboratories (Saint Germain sur l'Arbresle, France). The animals were randomly divided into groups of five and housed in a controlled environment (22°C, 12 h/12 h light/dark cycle, and ad libitum access to food and water). All animal experiments were performed in compliance with the guidelines of the Institut Pasteur de Lille Animal Care and Use Committee, the Amsterdam Protocol on Animal Protection and Welfare and Directive 86/609/EEC on the Protections of Animals Used for Experimental and Other Scientific Purposes (updated in the Council of Europe's Appendix A). Animal handling was also compliant with French legislation (the French Act 87-848, dated 19-10-1987) and the European Communities Amendment of Cruelty to Animals Act 1976. The study's objectives and procedures were approved by the regional Ethic and Welfare Committee for Experiments on Animals (Lille, France; approval number: 04/2011-019R).

### 2.4. *In Vivo* Experimental Design, Induction of Colitis, and Sampling

After one week of acclimatization, mice were administered intragastrically with 500 *μ*L/mice of selected fibers suspended in saline solution (final dose level: 4 g/kg of bodyweight) daily for either 5 days (the short-term treatment) or 3 weeks (the long-term treatment). Control mice received saline solution alone under the same conditions. Before the induction of colitis, feces were taken for microbiota analysis. Caecal content was also collected from healthy mice for SCFA assays. Samples were snap-frozen and stored at −80°C prior to analysis.

To induce a moderate level of inflammation, we used a standardized murine TNBS colitis model (described in [25]). Briefly, a 50 *μ*L solution of 110 mg/kg TNBS (Sigma) in 50% ethanol was slowly administered into the colon via a 3.5 French gauge catheter. Mice were weighed, bled from the retroorbital venous plexus, and were sacrificed 48–72 h after TNBS administration. Clarified sera were frozen and stored at −20°C until cytokine assays were performed. Colons were removed, washed, and opened longitudinally. Samples of the distal colon (0.5 cm of the inflamed area) were processed in RNA stabilization solution (RNA-later, Ambion, Life Technologies, Carlsbad, CA, USA) and stored at −80°C for later gene expression analysis. Colon samples (1 cm of the distal part) were fixed in 4% formalin for histological analysis. Colon segments were also removed for further myeloperoxidase (MPO) assays.

### 2.5. Inflammation Scoring

Murine IL-6 and serum amyloid A (SAA) protein levels were measured by ELISA using commercial antibodies (BD Biosciences Pharmingen, San Diego, CA, USA and Biosource International (Camarillo, CA, USA), resp.), with a lower limit of sensitivity of 15 pg/mL for IL-6 and 30 ng/mL for SAA. Opened colons were graded for inflammation (using the Wallace score) by two blinded observers [26]. Fixed colons were embedded in paraffin and histological analysis was performed on 5 *μ*m tissue sections stained with May-Grünwald Giemsa reagent. Tissue lesions were scored according to the Ameho criteria [27]. The degree of polymorphonuclear neutrophil infiltration of the distal colon was assessed by quantifying neutrophil granule MPO levels, as described earlier [28].

### 2.6. Gene Expression Analysis

Samples were crushed using the FastPrep Lysing Matrix D (MP Biomedicals, Santa Ana, CA, USA) and total RNA was isolated using RNA spin columns (Macherey-Nagel, Hoerdt, France). Reverse transcription and real-time PCR were performed with reaction kits (high capacity cDNA RT kit) and reagents (Universal PCR Master Mix) from Applied Biosystems (Courtaboeuf, France), according to the manufacturer's instructions. The PCR reactions were performed with a MX3005P Stratagene machine (Agilent Technologies, Massy, France). For the target genes Nos2, PPAR*γ*, IL-1*β*, Cox2, and IL-6, a custom gene expression assay (TaqMan, Applied Biosystems) was used with the commercially designed and validated primers given in Table 1. The housekeeping gene beta actin was run as a reference gene. At least ten biological replicates were measured for each exposure condition. The recorded data were analyzed using the 2^−ΔΔCt^ calculation method and expressed as a fold-increase over the values in control mice.

### 2.7. Pyrosequencing Procedures and Analysis

Fresh feces were collected at the start of the experiments and 3 weeks after the long-term fiber treatment. Samples were snap-frozen and stored at −80°C prior to nucleic acid extraction.

*DNA Extraction and PCR Amplification*. Nucleic acid was extracted as described previously [29]. 16S rRNA genes were amplified using PCR primers [30] targeting the V5 and V6 hypervariable regions. The forward primer contained the Titanium A adaptor sequence (5′-CCATCTCATCCCTGCGTGTCTCCGACTCAG-3′) and a barcode sequence. The reverse primer contained the Titanium B adaptor sequence (5′-CCTATCCCCTGTGTGCCTTG-3′). For each sample, a PCR mix of 100 *μ*L contained 1x PCR buffer, 2 U of KAPA HiFi Hotstart polymerase blend and dNTPs (Kapa Biosystems, Clinisciences, Nanterre, France), 300 nM primers (Eurogentec, Liège, Belgium), and 60 ng of genomic DNA. Thermal cycling consisted of initial denaturation at 95°C for 5 min, followed by 25 cycles of denaturation at 98°C for 20 s, annealing at 56°C for 40 s, and extension at 72°C for 20 s, plus final extension at 72°C for 5 min. Amplicons were visualized on 1% agarose gels with GelGreen Nucleic Acid gel stain in 1x Tris-acetate-EDTA (TAE) buffer and were then cleaned with the Wizard SV Gel and PCR Clean-up System (Promega, Charbonnieres les Bains, France), according to the manufacturer's instructions.

*Amplicon Quantitation, Pooling, and Pyrosequencing*. DNA amplicons and purified pooled DNA concentrations were determined using the Quant-iT PicoGreen dsDNA reagent and kit (Life Technologies), according to the manufacturer's instructions. Assays were carried out using 2 *μ*L of cleaned PCR product in a total reaction volume of 200 *μ*L in black, 96-well microtiter plates. Following quantitation, cleaned amplicons were combined in equimolar ratios in a single tube. The final pool of DNA was eluted in 100 *μ*L of nuclease-free water and purified using an Ampure XP Purification Systems, according to the manufacturer's instructions (Agencourt Biosciences Corporation, Beckman Coulter, Beverly, MA, USA) and resuspended in 100 *μ*L of TAE 1x buffer. Pyrosequencing was carried out using primer A on a 454 Life Sciences Genome Sequencer FLX instrument (Roche, Branford, CT, USA) using titanium chemistry.

*16S rRNA Data Analysis*. The sequences were assigned to samples as a function of their sample-specific barcodes. The sequences were then checked against the following quality control criteria [31]: (i) an almost perfect match with the barcode and primers (one mismatch/deletion/insertion per barcode or per primer was allowed); (ii) at least 240 nucleotides in length (not including barcodes and primers); and (iii) no more than two undetermined bases (denoted by N). Each pyrosequenced dataset that met these criteria was assigned to a family with the ribosomal database project (RDP) classifier (version 2.1, http://rdp.cme.msu.edu) with a confidence threshold >80%. The Chao richness estimate was calculated using Mothur software (for more details, see http://www.mothur.org/wiki/Chao).

### 2.8. Quantification of SCFA Production

The SCFAs acetate, butyrate, and propionate in cecal suspensions were assayed on a gas chromatograph (Shimadzu) using a capillary free fatty acid packed column (Achrom, Zulte, Belgium; 30 m × 0.33 mm × 0.25 *μ*m), a split injector, and a flame ionization detector. The results are reported in mmol per gram of wet caecal material.

### 2.9. Statistical Analysis

Experimental groups were compared with their respective controls in nonparametric one-way analyses of variance, Mann-Whitney *U* tests, or Student's *t*-test, as appropriate. The statistical significance of the results is denoted as ^∗^ *P* < 0.05, ^∗∗^ *P* < 0.01, and ^∗∗∗^ *P* < 0.001. Data are presented as the mean ± standard error of the mean.

## 3. Results

### 3.1. The Immunomodulatory Effect of Fibers on PBMCs

**Figure 1:**
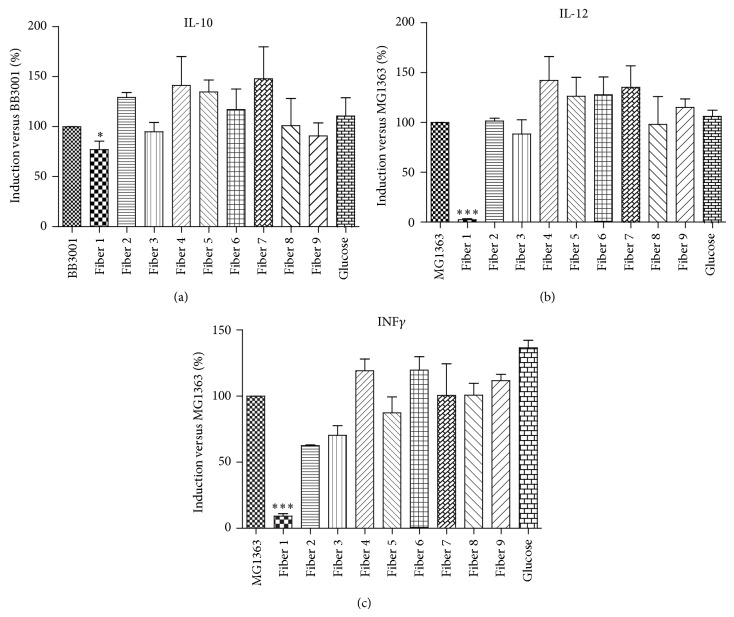
Immunomodulatory effects of nine different types of fibers: peripheral blood mononuclear cells were stimulated with control MG1363 strain or BB3001 strain, in order to induce a strong immune response. To demonstrate immunomodulatory effects, fibers were added at the same time as the bacteria. Levels of the cytokines IL-10 (a), IL-12 (b), and IFN-*γ* (c) were measured in the culture supernatant. Data are presented as the percentage induction relative to the control strains. ^∗^ *P* < 0.05, ^∗∗∗^ *P* < 0.001.

The fiber-induced production of cytokines by human PBMCs was investigated under baseline conditions and after bacterial stimulation. The nine fibers tested were not cytotoxic (as assessed by trypan blue staining) and did not induce the detectable production of cytokines under baseline conditions (data not shown). After bacterial stimulation, only Fiber 1 slightly inhibited the production of IL-10 (with a decrease of 23%, relative to the anti-inflammatory control strain* B. longum* BB3001). More importantly, Fiber 1 was associated with much lower levels of IL-12 and IFN-*γ* (with respective decreases of 98% and 89%, relative to the proinflammatory control strain* L. lactis* MG1363 (*P* < 0.001)) (Figure 1). This inhibition was dose-dependent (results not shown) and did not occur with any of the other fibers. These results show that Fiber 1 has a strong, specific, immunosuppressive effect.

### 3.2. The Effects of Long-Term Administration of Selected Fibers in the TNBS Colitis Model

On the basis of the* in vitro* results, we decided to study Fiber 1's strong immunosuppressive effect* in vivo*. Fiber 2 was used as a control. Mice fed for 3 weeks with the selected fibers (at 4 g/kg) did not display any bodyweight changes or any modulation of inflammatory gene expression (data not shown). We observed similar food intakes and normal stool consistency in all groups of mice.

**Figure 2:**
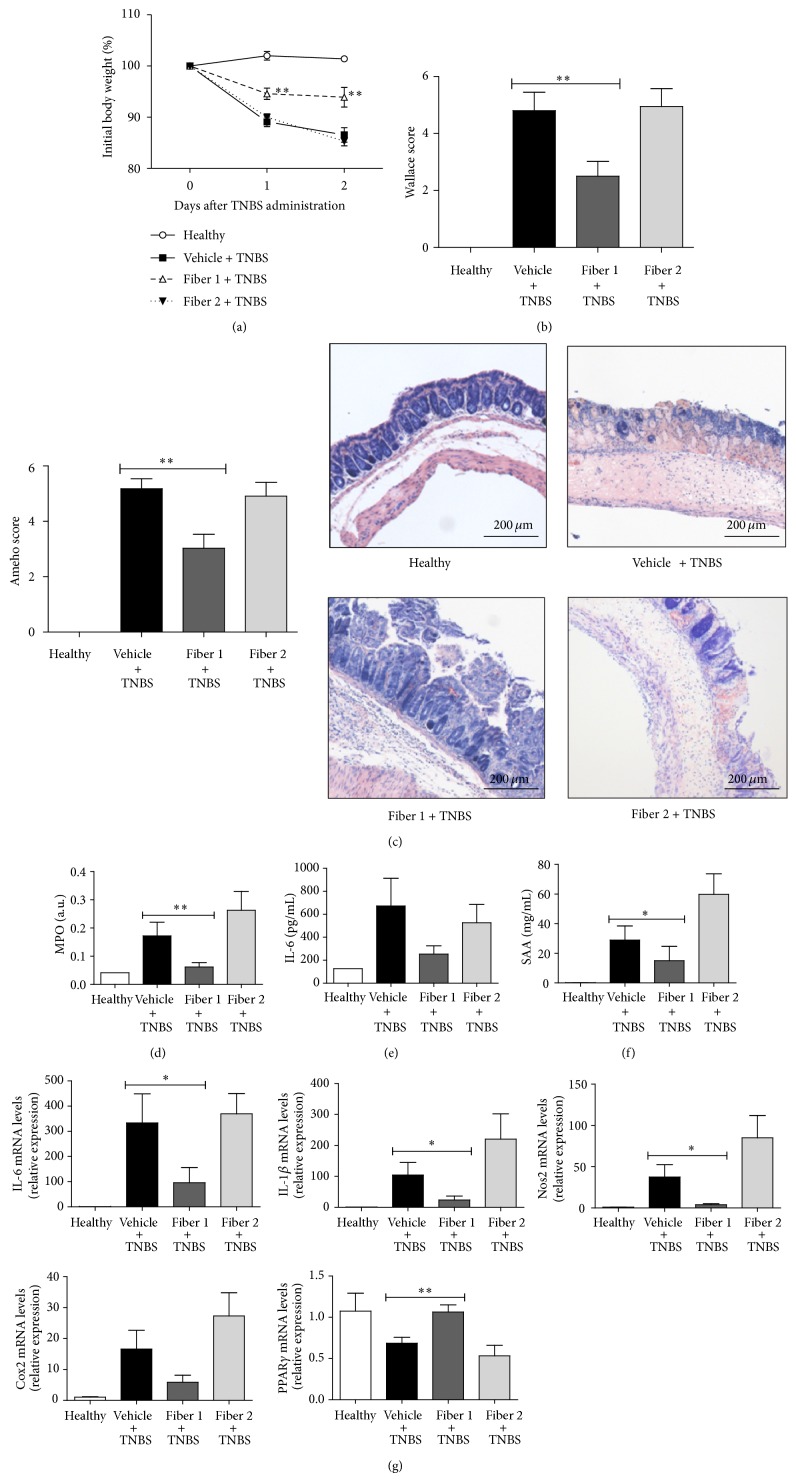
Effect of long-term administration of selected fibers in a TNBS colitis model: in addition to their standard diet, mice were administered water, Fiber 1, or Fiber 2 for three weeks. Next, TNBS colitis was induced. The body weight change (a), the macroscopic score (b), the histological score of representative May-Grünwald-Giemsa stained colon sections (c), the myeloperoxidase activity (d), serum levels of IL-6 (e) and SAA (f), and the expression of inflammatory genes in the colon (g) were scored. ^∗^ *P* < 0.05, ^∗∗^ *P* < 0.01.

To assess the effects of these two fibers with opposing effects on the immune response during colonic inflammation, we studied the TNBS murine model of inflammatory bowel disease. The clinical and histopathological features of the severe colitis induced by TNBS exposure resemble those seen in Crohn's disease. During the development of TNBS-induced inflammation, the mice had lost 13% of their initial body weight 2 days after the induction of colitis. A 3-week oral pretreatment with Fiber 1 (but not Fiber 2) restricted the weight loss significantly (to 6%; *P* < 0.01; Figure 2(a)). Compared with mice having ingested vehicle (phosphate buffer) alone, pretreatment with Fiber 1 was associated with a 48% relative reduction in the Wallace macroscopic inflammation score (4.8 ± 0.68* versus *2.5 ± 0.52, respectively, *P* < 0.01) (Figure 2(b)). This inhibition was confirmed in a histological assessment, which showed significantly less severe colonic damage (according to the Ameho score) in mice treated with Fiber 1. In contrast, pretreatment with Fiber 2 did not reduce the macroscopic and histological damage scores (Figure 2(c)). Consistently, MPO activity was significantly lower (by 65%; *P* < 0.01) in mice fed with Fiber 1, relative to control mice fed with Fiber 2 (Figure 2(d)). Likewise, there was reduction in serum IL-6 and SAA levels in mice administered with Fiber 1 (by 62% and 48%, resp.; *P* < 0.05; Figures 2(e) and 2(f)). Pretreatment with Fiber 2 was not associated with a significant change in serum IL-6 and SAA levels. We next studied the expression of colonic genes as markers of local inflammation. With Fiber 1 supplementation, we observed a significant reduction in expression of the inflammatory genes IL-6 and IL-1*β* and the oxidative stress genes Cox2 and Nos2 (all *P* < 0.05, relative to control mice). We observed also the restoration of PPAR*γ* expression (*P* < 0.01), which is known to (i) inhibit the expression of inflammatory cytokines and (ii) prompt immune cells to differentiate into anti-inflammatory phenotypes (Figure 2(g)). These changes in gene expression highlighted a reduction in the inflammatory state and suggest that only the oral administration of Fiber 1 has a significant inhibitory activity. This finding was confirmed by all the other inflammatory parameters measured in mice following TNBS colitis induction.

### 3.3. The Effects of Short-Term Administration of Selected Fibers in a TNBS Colitis Model

**Figure 3:**
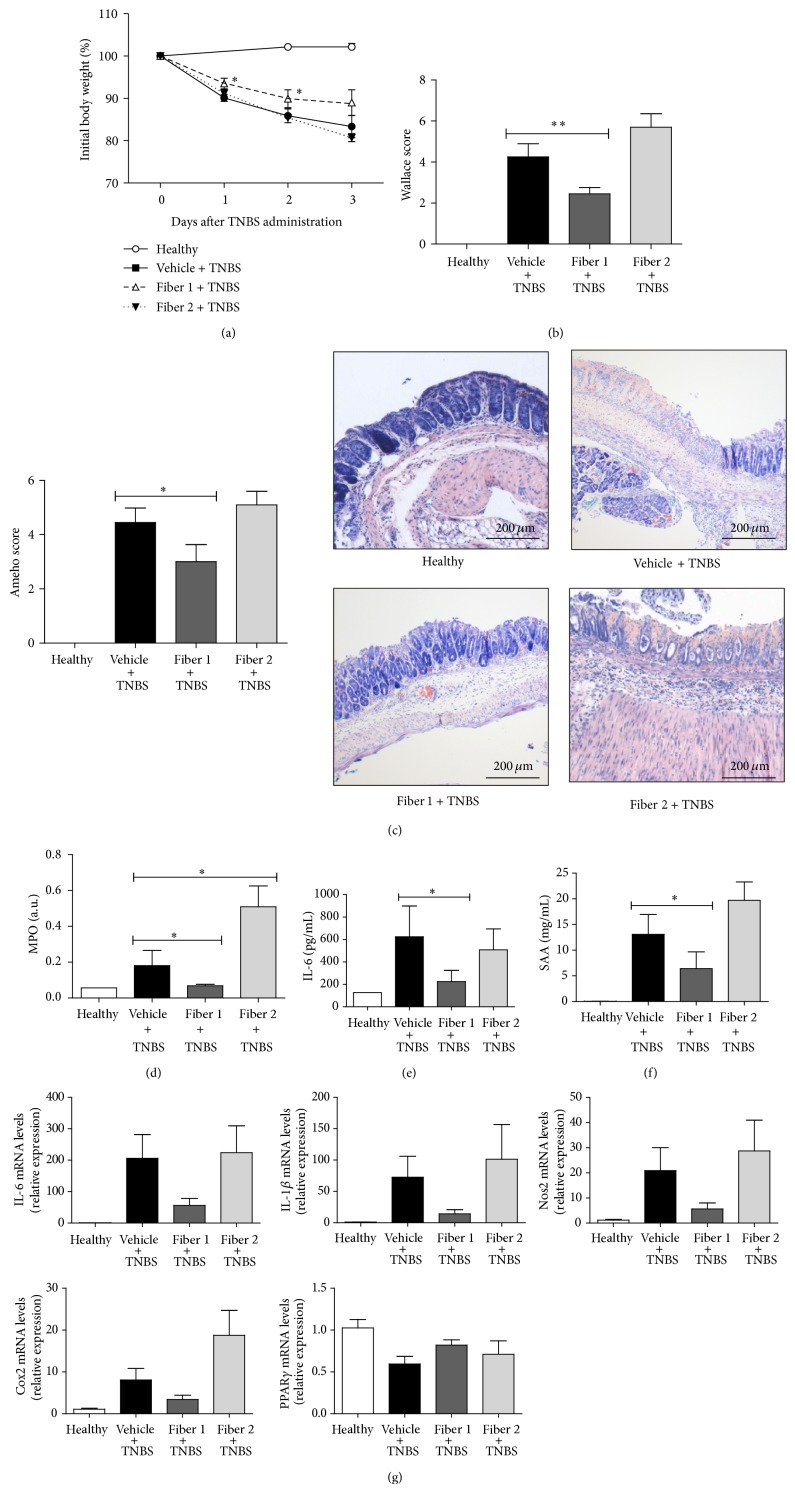
Effect of short-term administration of selected fibers in a TNBS colitis model: in addition to their standard diet, mice were administered water, Fiber 1, or Fiber 2 for five days. Next, TNBS colitis was induced. The body weight change (a), the macroscopic score (b), the histological score of representative May-Grünwald-Giemsa stained colon sections (c), the myeloperoxidase activity (d), serum levels of IL-6 (e) and SAA (f), and the expression of inflammatory genes in the colon (g) were scored. ^∗^ *P* < 0.05, ^∗∗^ *P* < 0.01.

To study the fibers' potential direct immune effects (rather than the established fermentation-mediated prebiotic effect), we pretreated mice with fibers for only 5 days. In this second experiment, we prolonged the colitis for an additional day (in order to obtain more intense colitis). Here, exposure to TNBS induced a progressive body weight loss of 18%. Short-term treatment with Fiber 1 limited the body weight loss to 13%, whereas Fiber 2 had no effect on this parameter (Figure 3(a)). In terms of macroscopic and histological scores, treatment with Fiber 1 was associated with a lower Wallace score (2.45 ± 0.31, versus 4.25 ± 0.64 in TNBS-treated control mice; *P* < 0.01) and a lower Ameho score (3 ± 0.63 and 4.45 ± 0.53, resp., *P* < 0.05) (Figures 3(b) and 3(c)), confirming the histopathological results. Levels of IL-6 and SAA were much lower (by 64% and 51%, resp.; *P* < 0.05 for both) in mice fed with Fiber 1. Likewise, MPO activity was lower (by 62%; *P* < 0.05) in mice fed with Fiber 1 than in TNBS control mice (Figures 3(d), 3(e), and 3(f)). The colonic expression levels of inflammatory gene markers were also lower in mice fed with Fiber 1, albeit not significantly (Figure 3(g)). All the results from this second TNBS experiment were consistent with the previous data and demonstrated a protective effect of Fiber 1 even when administered for just a few days.

### 3.4. Changes in Colonic Fermentation and SCFA Production after a Long-Term Treatment with Prebiotic Fibers

**Figure 4:**
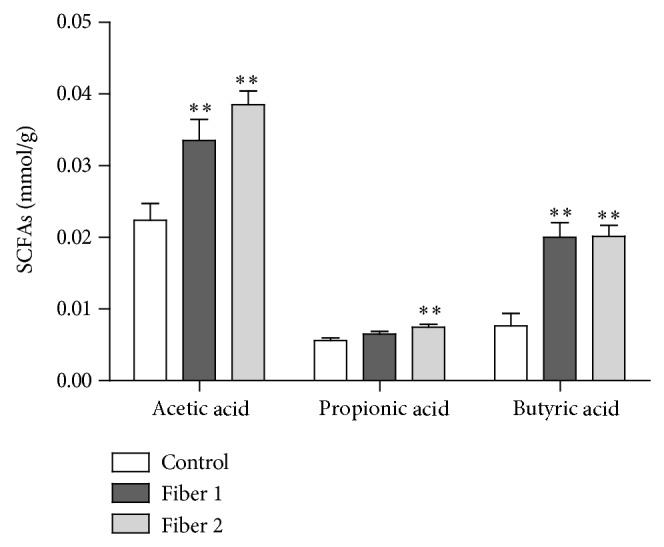
Colonic fermentation after 25 days of treatment with the selected fibers: levels of SCFAs (acetic acid, propionic acid, and butyric acid) were measured in the caecal samples of mice from the control, Fiber 1, and Fiber 2 groups after 25 days of treatment. Values are reported in mmol per gram of wet caecal material. ^∗∗^ *P* < 0.01.

To rule out the possibility that the observed protection against colitis was due to a “standard” prebiotic effect, we measured the production of anti-inflammatory SCFAs (i.e., the cecal SCFA content of mice administered Fiber 1, Fiber 2, or a control saline solution for 3 weeks). Long-term treatment with either Fiber 1 or Fiber 2 significantly increased levels of acetic acid (by 33% and 42%, resp.; both *P* < 0.01) and butyric acid (62% and *P* < 0.01 for both), relative to controls (Figure 4). Caecal propionic acid levels were only slightly higher in mice treated with Fiber 2 (26%, *P* < 0.01). The fibers' similar SCFA profile during long-term treatment suggests that protection in the TNBS colitis model is due to mechanisms specifically associated with Fiber 1, rather than to fermentation processes.

### 3.5. Modulation of the Microbiota during Long-Term Treatment

**Figure 5:**
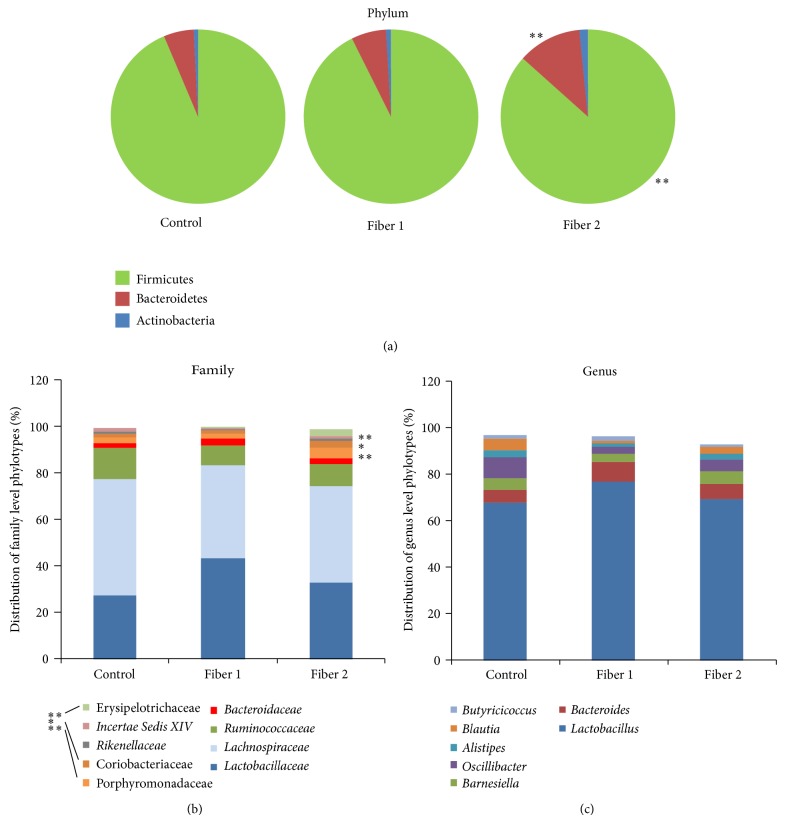
Distribution of bacterial phylotypes (a), families (b), and genera (c) in the fecal pellets of mice treated with Fiber 1, Fiber 2, or water alone (control). ^∗^ *P* < 0.05, ^∗∗^ *P* < 0.01.

Analysis of the fecal microbiota was carried out via 454 pyrosequencing of the V5-V6 region of the 16S ribosomal RNA. With operational taxonomic unit (OTU) cut-offs of 0.03, 0.05, and 0.10, the samples from the control, Fiber 1, and Fiber 2 groups did not differ significantly in terms of biodiversity (as assessed by the nonparametric Shannon index of diversity). However, with an OTU cut-off of 0.03, Fiber 1 and Fiber 2 treatments significantly improved microbial richness; the Chao richness index was 2936 ± 171 in control mice, 3632 ± 196 in mice fed with Fiber 1 (*P* = 0.0206), and 3717 ± 240 in mice fed with Fiber 2 (*P* = 0.0130). Treatment with Fiber 2 was also associated with minor changes in the composition of the fecal microbiota at both the phylum and family levels. In fact, a 25-day treatment with Fiber 2 induced a small but significant increase in the number of Bacteroides and a significant decrease in the number of Firmicutes (Figure 5(a)). At the family level, we observed a slight increase in the numbers of Erysipelotrichaceae, Coriobacteriaceae, and Porphyromonadaceae (Figure 5(b)). In contrast, Fiber 1 did not have a significant effect on the bacterial composition, which might explain the difference in protection between the two fibers. Similarly, the distribution of bacterial genera in the distinct groups of mice was barely affected. Consequently, the specific protection provided by Fiber 1 did not seem to be due to modulation of the microbiota, as could have been expected after the prolonged administration of a prebiotic. A direct effect of the fiber on the immune system thus appears to be more likely in this context.

## 4. Discussion

The present study aimed to characterize a potential direct effect of carbohydrate-based low-digestible fibers on immunity in* in vitro* and* in vivo* models. In view of the complex relationships between diet, intestinal microbiota, their metabolites, and the host immune system, the intrinsic immunomodulation induced by different food compounds (including dietary fibers) acts additively or synergistically with well-characterized prebiotic effects [18, 32]. The recent finding that “nontraditional prebiotic mechanisms” may contribute to the immunomodulatory effects of bacteria-derived exopolysaccharides has attracted much attention [33–35]. For example, it is well known that prebiotics can inhibit the adhesion of pathogenic bacteria* in vitro* to human epithelial cells and thus act as decoy receptors [36]. This is also the case for selected commensals or probiotic bacteria [20], known to indirectly influence subsequent immune signaling in this way. As previously suggested [37], prebiotic oligosaccharides might prevent cell activation and therefore the induction of inflammatory responses by mimicking binding sites on immune cell surfaces.

This mechanism might be involved in our study, since we observed that administration of one particular fiber (Fiber 1) inhibited the expression of both pro- and anti-inflammatory cytokines. Both epithelial and immunocompetent cells might be targeted by this type of carbohydrate, since some prebiotics can diffuse through epithelial monolayers and potentially modulate immune cells located in the subepithelial environment [38]. Moreover, it was recently shown that low- or nondigestible oligosaccharides can interfere with intestinal cell lines by activating the TLR4-NF*κ*B pathway [22]. Thus, prebiotics are ligands in intestinal cells that could compete with lipopolysaccharide signaling. Another hypothetical mechanism relates to the activation of TLR4 on monocytes, the activation of NF*κ*B, and the production of cytokines. Furthermore, it was recently demonstrated that prebiotics such as inulin, fructooligosaccharides (FOS), and glucooligosaccharides can act as direct or indirect TLR4 modulators by specifically upregulating TNF-*α*, IL-6, IL-17, IFN*γ*, and/or IL-10 in mice splenocytes or rat PBMCs [39]. In our study, the fructose-based fibers 6 and 9 did not produce this type of effect. Similarly, a specific *β*-glucan receptor (dectin-1) is reportedly involved in the nonopsonic recognition of zymosan on immune cells [40]. Lastly, by using an* in vitro* model in which dietary fibers (galactooligosaccharides, inulin, arabinoxylan, and glucan) were incubated with differentiated colonic epithelial cell cocultures, it was found that dendritic cells exposed to the spent media released regulatory cytokines [41].

In the present study, we identified two glucooligosaccharide fibers with different immunomodulatory properties* in vitro* and* in vivo*. However, their effects on SCFA fermentation and the gut microbiota composition were similar. Short-chain fatty acids (including butyrate) may fuel colonocytes, strengthen the epithelial mucosa, also activate FFAR pathways, and induce immunomodulation. Since long-term treatment with Fiber 1 had a greater effect than short-term treatment, one might expect Fiber 1 to have a prebiotic effect (mediated by the SCFA production) on the protection and/or modulation of the microbiota [15]. However, this effect was not sufficient* per se* to explain the observed anti-inflammatory effects of Fiber 1, since short- and long-term treatments with the control Fiber 2 did not provide protection but were associated with the generation of similar amounts of SCFAs.

Inflammatory bowel disease (most commonly Crohn's disease and ulcerative colitis) is a chronic, disabling condition with an increasing incidence in southern Europe. Although the etiology of IBD is unknown, the characteristic, disproportionate inflammatory response in the gut may develop through multifactorial cellular and subcellular mechanisms, involving genetics, the environment, dietary habits, and the composition of the gut microbiota [42]. Consequently, a range of treatments are used to reduce the symptoms and certain causes of IBD, although an exacerbated immune response can be directly targeted by immunosuppressive drugs (such as corticoids, 5-aminosalicylic acid, and anti-TNF-*α* antibodies), all having marked long-term side effects. Other strategies are based on microbiota-based dietary interventions, either by the use of prebiotic fibers, probiotics, or (most recently) fecal transplants of healthy microbiota.

Reduced bacterial diversity has been implicated in the mechanism of IBD [43]. Specific pathogenic/colitogenic bacteria (such as enteroinvasive* E. coli* or strains of* Fusobacterium*) and the lack or decrease in certain beneficial species (such as* Faecalibacterium prausnitzii* or* Roseburia hominis*) are reportedly linked to the development of IBD [44]. Hence, increasing overall microbial diversity with prebiotics may be one way of treating these pathologies. In the present study, treatment with various prebiotic fibers was associated with a slight increase in bacterial richness. However, this increase in microbiota diversity alone was not sufficient to explain the observed effect; the prebiotic Fiber 2 did not significantly affect the intensity of colitis.

In view of (i) the crucial role of carbohydrate-based prebiotic fibers in the management of gut homeostasis, (ii) the intricate relationships between immunity (via the gut associated lymphoid tissue, GALT, the regulatory T-cells, and the dendritic cells), (iii) the gut microbiota composition, and (iv) the corresponding metabolic pathways (SCFAs and their related receptors), our results in a TNBS model for colitis may have consequences for other diseases, notably overweight, obesity, and type 2 diabetes. Indeed, a number of metabolic disorders have been successfully treated by the administration of dietary fibers like FOS [45, 46]. Since obesity is characterized by a low-grade inflammatory state, an additional direct impact of dietary fibers on the immune response may be of major interest in the treatment of metabolic syndromes [47]. Moreover, a direct impact of dietary fibers on the migration of systemic immunocompetent cells can occur at sites remote from the gut [48].

In conclusion, our study results strongly suggest that intrinsic, nonprebiotic-driven effects of selected oligosaccharide and polysaccharide fibers can influence immunomodulatory functions. These fibers could be used to extend the well-known prebiotic mechanisms (i.e., better colonic fermentation and SCFA production) and enhance dietary interventions for the treatment of inflammatory disorders such as IBD, IBS, and other diseases with an immune component [6]. The use of fibers alone or in combination with selected probiotics (symbiotic preparations) could be considered. The observed effects of Fiber 1 may also explain how (i) fiber intake reduced inflammatory plasma markers in certain epidemiological studies and (ii) fiber supplementation in clinical studies impacts on inflammation through mechanisms unrelated to bodyweight [49]. Further studies are required, however, to define the structural basis of a direct effect of carbohydrate polymers in relation to signaling and receptor binding and investigate the underlying extraintestinal impact on regulatory cells involved in the immunoregulatory effect.

## Figures and Tables

**Table 1:** The list of target genes and the corresponding primer accession numbers.

Gene name	Abbreviation	Commercial reference
Actin beta	ActB	Mm 01205647_g1
Nitric oxide synthase 2 (inducible)	Nos2	Mm 00440502_m1
Peroxisome proliferator activated receptor gamma	Pparg	Mm 01184322_m1
Interleukin 1 beta	Il1b	Mm 01336189_m1
Prostaglandin synthase 2	Ptgs2 (Cox2)	Mm 00478374_m1
Interleukin 6	Il6	Mm 00439614_m1
